# The Review of Selected Non-Pneumatic Tires Properties—Mechanical Properties: Radial, Longitudinal, Lateral Stiffness, Rolling Resistance, Contact Path; Vehicle Applications

**DOI:** 10.3390/ma18174107

**Published:** 2025-09-01

**Authors:** Marcin Żmuda, Jerzy Jackowski

**Affiliations:** Faculty of Mechanical Engineering, Military University of Technology, 00-908 Warsaw, Poland; jerzy.jackowski@wat.edu.pl

**Keywords:** non-pneumatic tire, NPT stiffness (radial, lateral, longitudinal), contact path, rolling resistance, NPT vehicle applications

## Abstract

Nowadays, attempts to commercially apply non-pneumatic tires (NPTs) in a wide range of vehicles can be observed. These types of wheels use a specific structure and material selection to mimic the function of compressed air in pneumatic tires (PTs). This paper reviews the mechanical properties and vehicle applications of non-pneumatic tires. This review will provide information about the influence of the shape of the radial, longitudinal, and lateral characteristics, as well as the possibility of selecting contact pressure values at the wheel design stage and the influence of the type of elastic structure on the concentration of pressures in the contact length. The radial characteristics of non-pneumatic tires depend on the type of elastic structure. The spoked elastic structure will be characterized by greater displacements compared to the cellular structure, which will reduce radial stiffness. The radial stiffness of non-pneumatic tires is increased by increasing the number of elastic structure elements and their thickness and decreasing their length. The longitudinal stiffness of non-pneumatic tires increases with the number of elements forming the elastic structure and with the elastic structure’s lack of susceptibility to circumferential deformation. Spoked non-pneumatic tires will have lower longitudinal stiffness compared to cellular non-pneumatic tires. The elastic structure is characterized by a low susceptibility to lateral deformation, which contributes to the high lateral stiffness of the non-pneumatic tire. Non-pneumatic tires have a limited ability to shape the contact patch parameters, which mainly depend on the vertical load and the shape of the tread area. The type of band used will influence the formation of contact pressures. An isotropic elastomer used in a shear band will cause pressure concentration at the ends of the contact length. A more uniform pressure distribution is achieved by using a laminated elastomer.

## 1. Introduction

This article is the second part of a literature review analyzing NPTs. The first part [1] includes literature analysis methodology, detailed analysis of the structure of individual NPT components (rim, elastic structure, shear beam/band with tread) and their influence on selected NPT features, and material guidelines/examples for individual NPT components based on patent applications analysis. This review presents information on radial stiffness, longitudinal stiffness, lateral stiffness, rolling resistance, and contact path. Radial stiffness is one of the most important features of wheels. Its curve will affect the range of loads transferred to the vehicle (suspension and supporting structure) as a function of wheel deflection [2]. The radial stiffness characteristic expresses the force acting on the wheel axis (in the normal direction) as a function of the wheel axis displacement (wheel deflection). The radial stiffness characteristic is determined in quasi-static or dynamic conditions (for a non-rolling and rolling wheel) [2,3]. The radial characteristic is an important feature of tires that affects wheel vibrations, smoothness of driving, interactions with the road surface, rolling resistance (energy losses related to wheel deformation), and values of loads transferred to other vehicle elements (resulting from the course of the characteristics and deflection of the wheel). In the case of pneumatic tires, their radial stiffness can be formed by changing the inflation pressure.

The longitudinal and lateral (tangential) stiffness of the tires plays an important role in their dynamics and describes the susceptibility of the wheel to deformations in the tangential direction. In the steady-state vehicle motion, the tangential reactions of the wheels depend mainly on their slip. In the transient state, the pneumatic tires need to travel a certain distance to increase the horizontal deflections. Tangential stiffnesses will affect the relaxation length; it means a distance to increase the tangential deflections, where tangential force obtains a constant value [4]. The relaxation length reflects the longitudinal and lateral flexibility of the tire carcass [3,4].

The lateral force acting on the wheel affects the vehicle’s steerability in curvilinear motion. Displacements of the wheel’s plane in curvilinear motion and lateral deformation of the wheel’s side walls occur under the influence of lateral forces. The lateral stiffness characteristic provides information on the influence of the wheel used on the vehicle’s stability and steerability [2,3,4,5]. Too low a value of the lateral stiffness of the wheel may negatively affect the directional stability of the vehicle, while wheels with too high lateral stiffness will be susceptible to slipping.

The basic parameters describing the interaction of the NPT with the non-deformable ground are contact pressure and contact patch (described by length, width, surface area, and shape). The width of the contact patch is strictly dependent on the shape of the tread face, which has been demonstrated in experimental studies [6]. The shape of the contact patch significantly depends on the deformation possibilities of a wheel/tire element’s main components, i.e., the side, shoulder, and tread face. The NPT, characterized by an “arc-shaped” tread face, increased the width of the contact patch with the increase of the vertical load. Changing the load of the wheel equipped with a “flat” tread face had a slight effect on the change in the width of the patch. In the case of pneumatic tires, the cord threads of the carcass (which is the load-bearing element of the tires) are vulcanized across the entire cross-section of the tire, i.e., from one bead to the other, which allows for deformation of the tire shoulder and sidewall depending on the inflation pressure and the type of carcass (radial, diagonal).

Rolling resistance is one of the most important parameters for evaluating tire properties and the interaction between tires and road surfaces. It encompasses energy losses associated with wheel deformation and phenomena transferred within the wheel-surface interaction area [7]. The volume of the wheel section that undergoes deformation and the extent of the deformation significantly impact energy losses. This issue is particularly important for NPTs, which usually have a thicker band.

Section 2 summarizes the most important explanations of selected terms used in the article, which may cause difficulties in understanding without reading the first part of the literature review.

Section 3 presents methods of shaping selected NPTs’ characteristics. Attention is focused on the radial stiffness characteristic, lateral stiffness characteristic, longitudinal stiffness characteristic, contact patch with non-deformable ground, and rolling resistance.

Section 4 provides information on the use of NPTs in wheeled vehicles.

## 2. Definitions

**Non-pneumatic tire (NPT)**—a type of wheel that transmits vertical load and traction forces from the road to the vehicle and also provides directional control of the vehicle, and to provide these functions, the wheel does not require the maintenance of gas or fluid (especially under a specific pressure) [8].

**Top loader**—a type of vertical load transmission mechanism used in wheels. Vertical loads are transmitted mainly by wheel components, which expand as a result of the vertical load. The components are generally located in the part of the wheel outside the contact zone [9].

**Bottom loader**—a type of vertical load transfer mechanism used on wheels. Vertical loads are transmitted mainly by compressing the part of the wheel that is located between the contact zone and the wheel’s axis [9].

**Shear beam/band**—an annular bendable part of the NPT to which an elastic structure is attached on the inside. On the outside of the band is a tread, which is usually firmly connected to the band. The band is made as reinforced or unreinforced [9].

**Reinforced shear beam/band**—consists of at least one layer of reinforcement made of non-stretchable material, e.g., cord fabric. If more than one layer is used, the material (elastomer) between the reinforcements is called the core [9,10,11].

**Core of shear beam/band**—occurs in the reinforced band. It is subjected to shear stress. The core can be divided into solid homogeneous (one elastomer), solid composite (elastomers with different properties in layers) [12].

**Unreinforced shear beam/band**—a band in which no reinforcement layer is used. Typically, this part of the NPT is made using an isotropic or composite elastomer [13] or webbing geometry/structure, i.e., a band with through-holes of a specific shape [14].

**Elastic structure**—the elastic-damping connection of the NPT rim to the shear beam/band [10,15,16]. It can be terminated with an outer and inner ring to facilitate the connection to the rest of the NPT parts.

**2D elastic structure**—a type of elastic structure formed by cutting a specific shape in the axial direction of the NPT. In the side view of the NPT, empty spaces are visible [1].

**3D elastic structure**—a type of elastic structure where individual elements of the elastic structure are interconnected in three-dimensional space [17].

**Spoked elastic structure**—a type of elastic structure in which individual elements are used to connect the rim to the band. Individual elements are radially distributed around the circumference of the NPT rim and do not decussate with other individual elements, mimicking “spokes” [16].

**Cellular/layered structure**—a type of elastic structure composed of individual cells located on a given layer. Cells have repeating shapes on a specific layer [15].

**Excess length** $L_{E}$—parameter used to describe the curvature of a spoked elastic structure, defining the difference in % between the spoke’s length in an undeformed state and the smallest distance between the spoke attachment points to the length of the undeformed spoke. Parameter influencing the curve of the radial stiffness characteristic [16].

## 3. Forming Selected NPT Characteristics

The mechanical properties of the NPT directly affect the vehicle’s movement on the road and have a significant impact on vehicle performance and include static and dynamic properties. A review of selected mechanical properties of NPT is presented in [18].

### 3.1. Radial Stiffness

**Figure 1:**
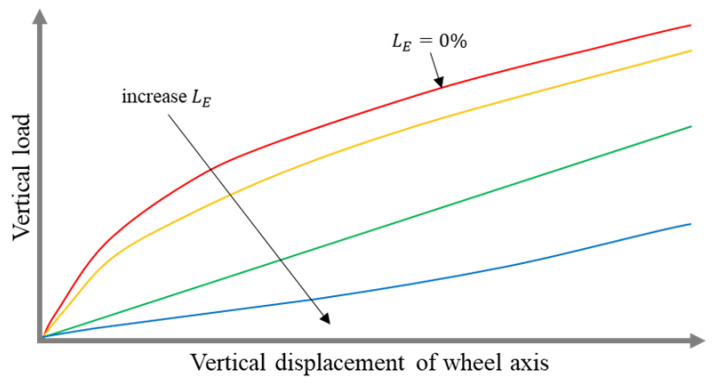
The influence of the spokes’ curvature of the elastic structure (excess length $L_{E}$ parameter) on the course of the radial characteristic (figure based on [16]).

During the exploitation of commercially available NPTs, the user has no possibility to change the radial stiffness characteristic, and its course is significantly influenced by the shear beam/band and elastic structure [10,15,16]. The radial stiffness of NPT equipped with single spokes is designed, e.g., by selecting their curvature. The curvature of the spokes is expressed by the excess of their length (parameter $L_{E}$ described in Section 2). The equation describing this relationship is presented in [1,16]. By increasing spoke excess length, the initial course of the radial stiffness characteristic will change. The NPT’s elastic structure with straight spokes, where the excess of their length is 0%, will be characterized by an initial degressive course [16]. Increasing the spokes’ curvature will affect the changes in the course of radial stiffness (Figure 1). Small displacements of the NPT axle with radially distributed straight spokes (especially in the initial radial characteristic) will cause a significant increase in the vertical force. The force will in turn be transmitted to the vehicle suspension elements. The opposite behavior can be observed for wheels with increased spoke curvature of the elastic structure.

Single radially arranged spokes ($L_{E}$ = 0%), whose attachment points to the inner and outer rings lie on straight lines passing through the NPT axle, will additionally contribute to high initial stiffness resulting from the lack of buckling susceptibility, which was observed in the research presented in [19].

The shape of the elastic structure should ensure its susceptibility to buckling in the part under the NPT axle. In [20] a review of patent solutions concerning the method of forming the NPT’s elastic structure in order to ensure its susceptibility to buckling is presented. In the case of an elastic structure consisting of single elements, the aforementioned susceptibility is achieved, e.g., by defining the curvature of the spokes. Spokes with a single curvature affect the significance of the displacement of the NPT axle, which is proportional to the value of the $L_{E}$ parameter [19]. The use of the elastic structure with several curvatures is more efficient because it will reduce the length of a single element (spoke) in comparison to a solution with a single curvature while simultaneously maintaining the same course of radial stiffness [16]. The radial stiffness of the NPT equipped with a spoke structure will increase with the increase in the number of spokes and their thickness and with the decrease in the curvature of the spokes [21].

**Figure 2:**
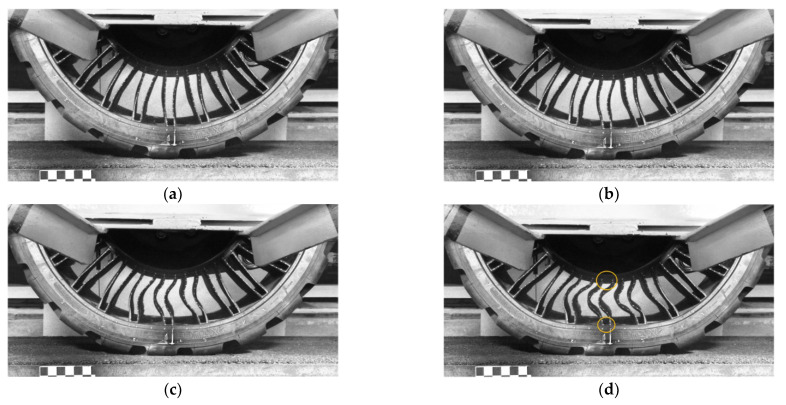
The band and the elastic structure under the NPT axis loaded by vertical force: (**a**) 0 N, (**b**) 5000 N, (**c**) 15,000 N, (**d**) 25,000 N (orange circle—spokes connected “perpendicular” to the inner and outer elastic structure rings that allow low values of stress).

Figure 2 shows the deformation of the band and elastic structure of an NPT designed for a skid-steer loader, the axis of which was loaded with a vertical force during the determination of the radial characteristic. The elastic structure of the analyzed NPT consisted of 25 pairs of spokes. The unloaded NPT located above the measuring surface is shown in Figure 2a. With the increasing load, the number of buckled spokes increases and the band “flattens” increasing the length of the contact patch. The spokes located directly under the wheel axis buckle the most. In the NPT, large values of the spoke attachment angles to the outer and inner rings were used, which can be particularly observed when analyzing the deformation of the spokes in Figure 2d (orange circles), which will influence low values of stress during spoke bending and tensioning.

**Figure 3:**
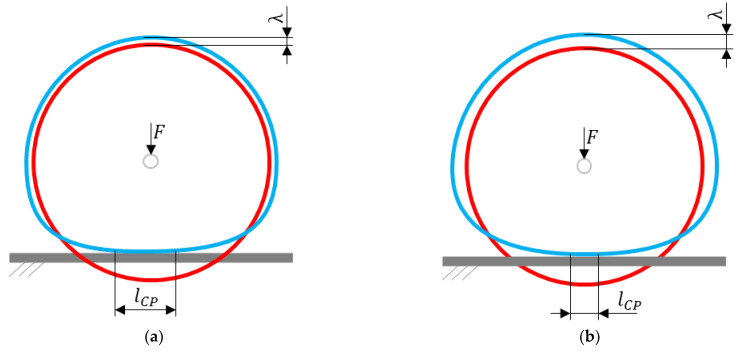
Shear beam/band deformation due to: (**a**) low counterdeflection stiffness, (**b**) high counterdeflection stiffness (figures based on [9,10]) (red color—undeformed NPT band shape, blue color—deformed NPT band shape, F—normal force, λ—counterdeflection, $l_{CP}$—contact path length).

The radial stiffness of the NPT (especially with an elastic structure with single spokes) is the resultant of the part of the NPT located in the immediate vicinity of the contact with the road and the upper part of the NPT located above the axis of rotation. When determining the radial stiffness characteristics for the NPT, the vertical displacement of the wheel axis and the stretching of the elastic structure λ located above the NPT axis are measured [10] (Figure 3). The circumferential compressive stiffness of the band will counteract compressive and bending loads to maintain an undeformed annular shape. The deformation λ (counterdeflection) is proportional to the applied vertical load. The counterdeflection stiffness $k_{\lambda }$ is defined as the ratio of the vertical force to the deformation λ [22]. Reducing the counterdeflection stiffness causes an increase in the vertical displacements of the band, which additionally affects the increase in the contact path length [10]. The low stiffness $k_{\lambda }$ of the spokes facilitates band deformation in the part above the wheel axis, influencing the shortening of the contact patch length. The $L_{E}$ parameter will have a significant influence on the range of wheel axis displacement, i.e., the wheel axis can move vertically until the spoke curvature is “straightened”. The counterdeflection stiffness $k_{\lambda }$ can be modified in many ways, e.g., by changing the values of the spoke elasticity modulus; the spoke length, spokes’ curvature and thickness, band diameter, band thickness and width [10,22].

The influence of radial and counterdeflection stiffnesses on band deformation and contact patch length can be described by the ring inextensible model [23]. The developed model finds application in radial pneumatic banded tires and NPTs and allows for the prediction of radial stiffness based on the measurement of selected wheel parameters.

**Figure 4:**
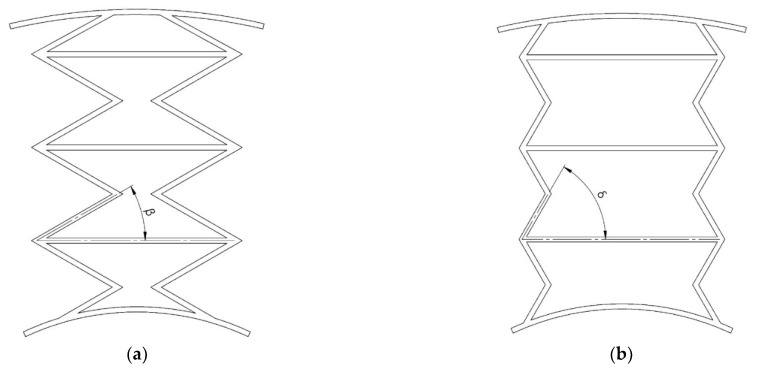
Auxetic cellular NPT structure: (**a**) with β internal angle, (**b**) with δ internal angle (figures based on [24]).

The displacement of the wheel axis equipped with a cellular structure will depend on the angles used in the cells. In [24], the change in the angle of the acoustic cells was analyzed. The displacements of the NPT axis decreased when the angle inside the cell increased (Figure 4). In the analyzed case, the δ angle is a multiple of the β angle.

**Figure 5:**
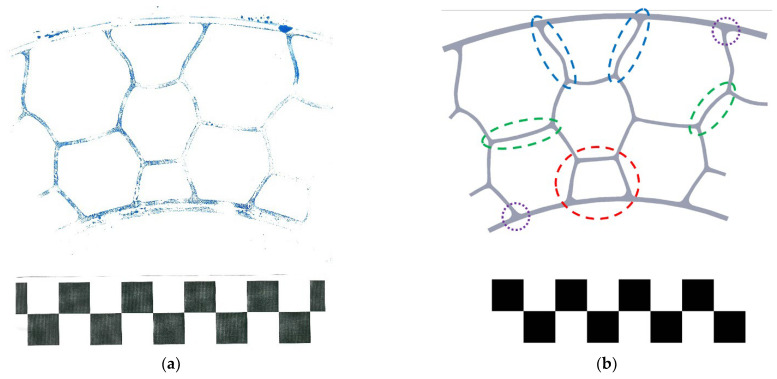
Shaping of the elastic cellular structure to ensure buckling susceptibility: (**a**) imprint of the NPT’s elastic structure, (**b**) model after processing.

Figure 5 shows the shape of the layered elastic structure of one of the NPTs available on the market. In order to reproduce the shape of the cellular structure, its side part was covered with ink, and then a sheet of paper was pressed (Figure 5a). During scanning, a checkerboard consisting of white and black squares was used for calibration during image processing. The NPT cell structure after image processing is shown in Figure 5b. The places where the layered structure connects to the outer and inner rings are of increased thickness (purple line), which may be due to the need to protect the structure from stress. The sides of the cell closest to the center of the NPT (red line) are straight sections. In the next layer, it can be seen that the sides of the hexagonal cells (green line) are built of arc sections. The sides of the cells located closest to the band and tread have an asymmetric curvature (blue line). Polygon elements, tangent to the circles concentric with the wheel rotation axis, will counteract the deformation of the remaining sides of the cellular structure, which will reduce the vertical displacement of the wheel and increase its stiffness compared to the spoke structure. This feature was observed in experimental studies [6]. In the experimental studies, the wheel with a cellular structure had higher stiffness compared to the wheel with a flexible structure of single spokes. In order to ensure the buckling susceptibility of the elastic cell structure, the following methods are used: cells with arcuate sides, asymmetry of the thickness of the cell sides, grooves/slots, or reduction in the thickness of the polygonal cell walls [20].

NPT radial stiffness will depend significantly on the elastic structure. In [25], the results of simulation research of the influence of a 2D cellular structure, which was named X-spoke, on radial stiffness were presented. Radial stiffness increased with the increase in the number of elastic structure elements and their thickness. An analogous relationship was obtained for the spoke structure [26]. The stresses in the elastic structure decreased mainly with the increase of the elastic structure elements and, to a lesser extent, with the increase of the spoke thickness.

In [27], the results of simulation research on NPT interaction with unevenness of rectangular cross-section were presented. The NPT was equipped with a 2D layered structure. The elastic structure, referred to as the rhombus structure, was created as a result of the intersection of single spokes consisting of straight sections located between the rim and the tread. During the determination of the radial stiffness characteristic with an unevenness located under the NPT axis, the absorbing properties of the analyzed NPT could be observed. When loading to the shortest unevenness, significant band deformations and significant contact pressures occurred in the contact with the unevenness. Increasing the length of the unevenness caused a decrease in band deformations, which was dictated by the fact that the unevenness covered a larger part of the NPT contact patch.

NPTs can be damaged, which may result from, e.g., stress concentration, load variation, or impulsive impacts. In [28], the influence of local damage on static and dynamic properties of NPT with a 2D cellular structure was analyzed using the finite element method. The influence of damage to the cell wall of the elastic structure on NPT was analyzed. The results of simulation research showed that damage to the cell wall in a selected circumferential position caused an increase in the shear beam stress and a decrease in radial stiffness.

In [29], the results of NPTs made by 3D printing research were presented. NPTs equipped with a 2D cellular structure were analyzed, taking into account different wheel positions (elastic structure) relative to the ground. Depending on the wheel position, different curves of the radial characteristics were obtained, which increased with decreasing the number of cell layers and the “density” of the elastic structure. The greatest differences were obtained for the structure with “large cells”, i.e., cells in which the external walls cover the inner and outer NPT rings.

Quick summary:

- Changing the inflation pressure of a pneumatic tire within the range specified by the manufacturer allows the radial stiffness of a typical pneumatic tire to be changed (and shaped). The radial stiffness of the NPT is defined at the design stage by selecting materials and shaping the elastic structure and shear band.
- Under vertical load, the elastic structure should deform in a predetermined manner to avoid sudden buckling. “Controlled” deformation is achieved by defining the curvature of the elastic structure elements or partially changing their thickness, as well as locating their ends (connections to inner and outer rings) on different lines passing through the axis of the wheel.
- The following solutions are used in NPTs to influence the formation of radial characteristics:
  ○ Spoke structure—reducing the number of spokes, their thickness, or increasing their curvature (increasing the length of the spoke) reduces radial stiffness.
  ○ Cellular/layered structure—the sides of the cells that are tangent to concentric circles with the NPT axis will limit the deformation of the other elements of the elastic structure, which will increase radial stiffness.
  ○ Increasing the susceptibility of the shear band to circumferential deformation decreases radial stiffness (as a result of increased NPT axis displacement).

### 3.2. Longitudinal Stiffness

**Figure 6:**
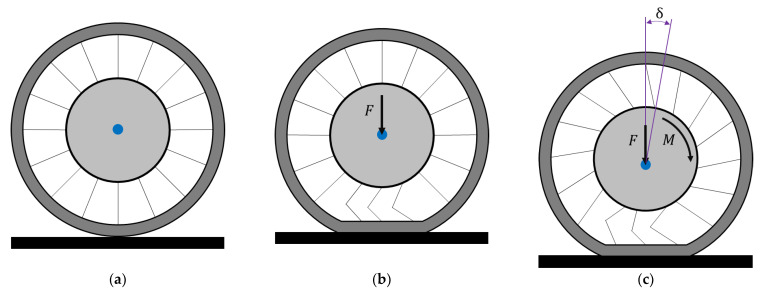
Behavior of elastic structure (spokes) under the influence of: (**a**) non-deformed NPT, (**b**) vertical load, (**c**) and external torque (figure based on [30]) (F—vertical force, M—external torque, δ—angular displacement, the blue dot marked the wheel axle rotation).

The elastic deformations in the circumferential direction of the NPT loaded with an external torque are the sum of the elastic deformations of the tread and the elastic structure. A certain angular displacement of the wheel rim relative to the band may also occur (Figure 6). In the case of a rolling NPT loaded with an external torque, the elasticity of the structure between the band and the rim may therefore cause a certain angular delay in the movement of the mentioned elements [30]. On this basis, it can be assumed that the elastic structure made of single spokes may be more susceptible to elastic circumferential deformations, especially if the attachment points to the inner and outer rings and the wheel axis lie in one line.

**Figure 7:**
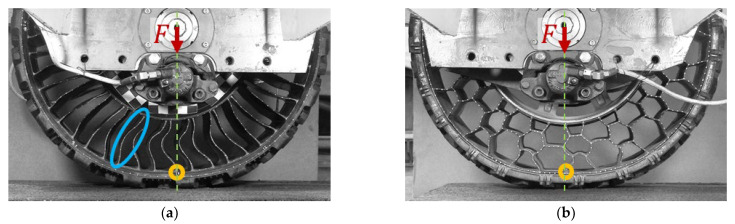

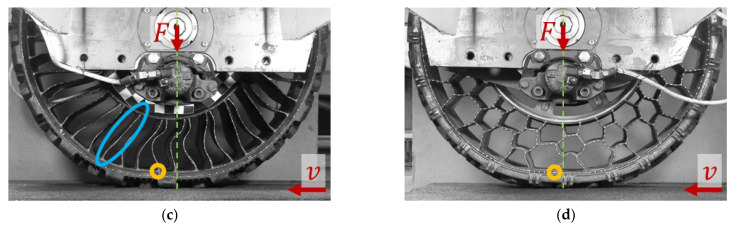
Deformation of the band and the elastic structure during the measurement of the longitudinal stiffness characteristic: (**a**) NPT with a spoke elastic structure loaded to the measuring track, (**b**) NPT with a cellular elastic structure loaded to the measuring track, (**c**) displacement of the measuring track relative to the spoke NPT, (**d**) displacement of the measuring track relative to the cellular NPT (F —vertical force, v—linear velocity of the measuring track, orange circle—the location of the analyzed connection point of the elastic structure with the band, green line—a straight line perpendicular to the measuring track passing through the NPT axis of rotation, blue ellipse—spoke behavior under loading).

In the case of NPTs, the type of elastic structure used affects their flexibility in the circumferential direction, which translates into the curved course of the longitudinal stiffness characteristic of the NPTs. Figure 7 shows the deformations of the NPTs’ spoke and layer structure used in Utility Terrain Vehicle (UTV) during the measuring of the longitudinal stiffness characteristic in quasi-static conditions (NPTs were vertically loaded to the measuring track and cannot rotate—brakes on). The orange circle marks the connection of the elastic structure with the outer ring located under the wheel axle. As a result of the movement of the measuring surface (track), the analyzed point moved. The spoke structure was more susceptible to longitudinal displacements than the analyzed cellular elastic structure. Deformations of the spoke elastic structure as a result of the movement of the measuring track are particularly easy to observe. The blue line marks one of the spokes, which buckled under the influence of a vertical load (Figure 7a) and stretched under the influence of a longitudinal force (Figure 7c). The cellular elastic structure is less susceptible to deformations in the longitudinal direction (Figure 7b,d).

The increased longitudinal stiffness of NPT with a spoke structure is obtained by, e.g., alternating the spokes, obliquely positioning the spokes in relation to the NPT axis (e.g., “zigzag” pattern) [10]. In [31], the NPT equipped with radial metal spokes, which were made as two- and three-joint hinges, was analyzed. The experimental research conducted there was aimed at assessing the effect of the number of joints and the number of spokes evenly distributed on the circumference, among others, on the static longitudinal stiffness characteristics. An increase in the value of stiffness was observed with the increase in the number of spokes and their joints. The decrease in the value of elastic deformations of the band as a result of the greater number of spokes was the cause of this phenomenon. It should be assumed that in the case of other NPTs (i.e., with elastomeric elastic structure) the nature of the described phenomenon will be similar.

The lack of susceptibility to circumferential deformations of the 3D printed NPTs with 2D elastic structures influenced the increase in longitudinal stiffness [29].

In [32], the results of static and dynamic research of an NPT with a spoked elastic 2D structure were presented. The spoke is a fragment of the Fibonacci spiral. The spokes’ connection points to inner and outer rings were located on different straight lines passing through the NPT axis. In the dynamic research, a different curve course of longitudinal force was obtained during braking and driving. The slip value and the maximum value of the longitudinal force occurred at 8% and 20% for the driven and braked wheel, respectively. After changing the spoke setting to asymmetric, the wheel obtained the same slip value (15%) in both directions. Reducing the spoke thickness affected the change in the course of the longitudinal characteristic force and reduced the longitudinal stiffness.

In [33], the results of simulation research of spoked NPT (with 2D layered structure) under multiple working conditions are presented. The numerical model of the wheel was validated with the results of experimental research conducted in static and dynamic conditions. The maximum longitudinal force was achieved for 10% slip, and the NPT camber had a small effect on the longitudinal force.

Quick summary:

- Compared to the spoke structure, the cellular structure limits the circumferential displacement of the shear band relative to the rim to a greater extent, resulting in greater longitudinal stiffness. This phenomenon is due to the greater number of elements forming the elastic structure.
- The lack of susceptibility to circumferential deformation of the elastic structure increases longitudinal stiffness.
- The asymmetry of the 2D elastic structure affects the circumferential stiffness of the driven and braked NPT differently.

### 3.3. Lateral Stiffness

The lateral stiffness characteristics of pneumatic tires significantly depend on the inflation pressure and the tire carcass and the reinforcements used. In most cases, NPTs do not have a sidewall. Lateral stiffness is shaped by the elastic structure located between the wheel rim and the band and by the susceptibility of the ring band to lateral deformations. The NPT is mostly characterized by a higher value of lateral stiffness in comparison to pneumatic tires, which was shown in comparative research [6]. The lateral stiffness of NPTs depends on the tread pattern and the type of cellular structure (made of elastomer) [34]. The use of an outer ring of appropriate thickness and an elastic cellular structure (especially its tangential elements) reduces lateral deformations of the NPT [15,35]. Analysis of the influence of the number of 2D elastic structure elements connecting the band with the rim revealed that the lateral stiffness decreases when the number of connecting elements is reduced [31].

**Figure 8:**
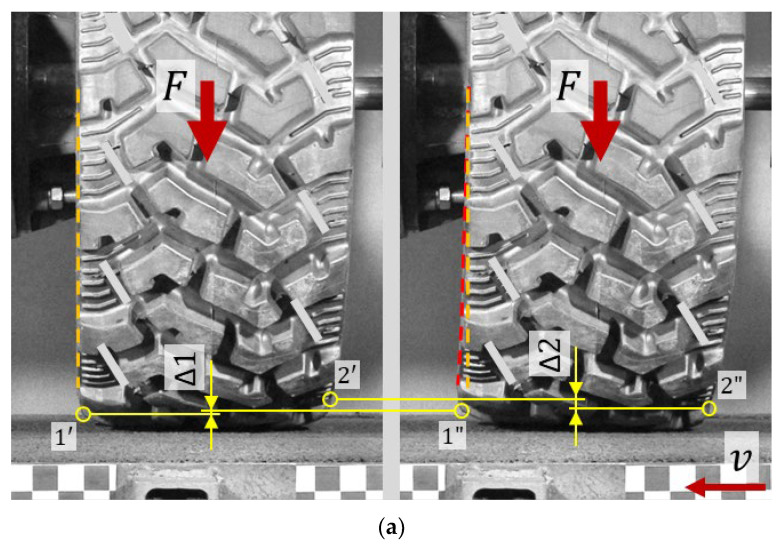

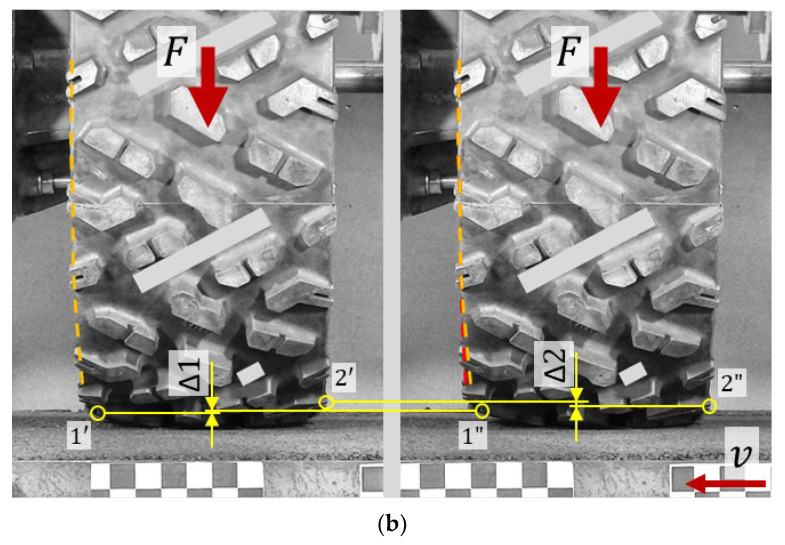
Deformation of the band and the tread during the measurement of the lateral stiffness characteristic: (**a**) NPT with a spoke elastic structure, (**b**) NPT with a cellular elastic structure (F—vertical force, v—linear velocity of the measuring track, 1′ 2′—analyzed points on the NPT loaded to the measuring track, 1″ 2″—analyzed points lying on the NPT loaded to the moving measuring track, yellow circles—the locations of the analyzed points of the tread, orange line—the line lying on the analyzed side of the loaded NPT to the measuring track, red line—the line lying on the analyzed side of the loaded NPT to the moving measuring track).

The deformation of commercially available NPTs during the measuring of lateral characteristics in quasi-static conditions is shown in Figure 8. The left part of the figure shows the NPTs loaded with the force F to the measuring track. The right part of the figure shows the NPTs, for which the measuring track additionally moves at the speed v. In order to identify the influence of the elastic structure on the range of displacements, points located on the side parts of the wheel were selected. Points marked as 1 moved upwards as a result of the lateral deflection of the NPTs, while points marked as 2 moved downwards. By observing the displacement ranges (Δ1, Δ2) of the analyzed points before (1′, 2′) and after the displacement of the measuring surface (1″, 2″), it can be observed that the NPT with the spoke elastic structure is more susceptible to lateral deformations. The cellular structure located under the wheel axle counteracts lateral deformations of the belt with the tread. The range of displacements can also be observed by analyzing the deformation of the NPT’s side before (orange line) and after displacement of the measuring track (red line).

Simulation research [36] have shown that as a result of the action of a lateral force, the contact patch changes from rectangular to triangular. This is related to the low capacity of the band or its lack of lateral deformation. As a result of the action of the lateral force, part of the band from the contact patch rises, causing a change in the shape of the contact patch. In this area, NPT mimics diagonal tires.

Currently, there is a lack of publications analyzing the lateral stiffness of NPTs equipped with the 3D elastic structure. It is anticipated that the structure can adequately mimic the properties in the lateral direction while providing the contact patch parameters of a radial tire.

Quick summary:

- NPTs equipped with the elastic 2D structure are characterized by greater lateral stiffness than pneumatic tires due to their lack of susceptibility to lateral deformation.
- Reducing the number of elements forming the elastic structure increases the lateral deformation range of NPTs, which reduces lateral stiffness.
- The elastic 3D structure can influence the ability to shape the desired lateral stiffness value.

### 3.4. Contact Path

**Figure 9:**
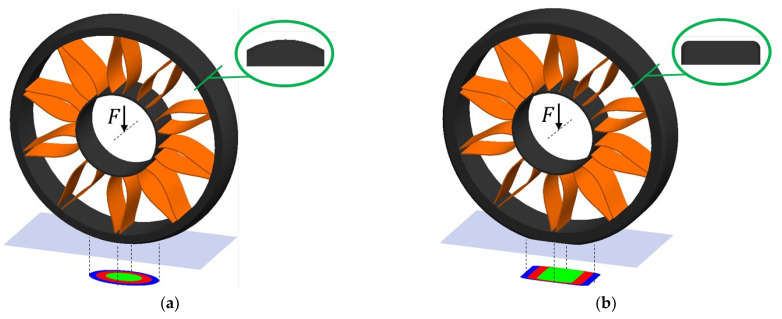
Effect of tread area shape on contact area: (**a**) NPT with flat tread face, (**b**) NPT with curved tread face.

The NPT tread is characterized by considerable thickness, which additionally makes its deformation difficult. The possibility of shaping the NPT contact path is presented in Figure 9, which shows a mental cutoff (mimicked by the gray surface) of a section of the. The NPT equipped with the “flat” tread area will be characterized by a contact patch close to rectangular (Figure 9a), while the NPT with the “arc” tread area will be characterized by an elliptical contact patch (Figure 9b). It is also worth noting that, in the case of NPT with a “flat” tread area, only the length of the contact path changes under the influence of increasing load. The green circles indicate the cross-section of the “flat” and “arc” treads with the band. The length of the contact patch will depend significantly on the density of the elastic structure, which will limit the deformation of the band in the contact area. The effect of the number of spokes on the deformation of the band with the tread under the influence of the vertical force is presented in [37]. The low density of the elastic structure caused an elongation of the contact patch and an undesirable arc deformation of the band with the tread.

The use of simplifications in numerical tests [38,39,40,41,42,43], i.e., a flat tread area without tread blocks or simplification of tread by modelling circumferential grooves, affects the obtaining of a rectangular NPT contact patch in the results.

The contact pressure on the non-deformable ground will be one of the NPT design criteria and will depend on the target group of vehicles (NPT size) as well as the method of making the band. For NPT with a uniform solid core, the product of the average value of the contact pressure and the radius of the outer reinforcement layer (which will be close in value to the NPT’s outer radius) will be equal to the product of the shear modulus of the elastomer layer of the shear beam core and its thickness [12,22]:

(1) $$P_{eff}R\approx Gh$$

$P_{eff}$–contact pressure (determined at the design stage),
G—dynamic shear modulus of the elastomeric layer of the uniform shear beam core,
h—thickness of the elastomeric layer of the shear beam core,
R—radius of the outer reinforcement layer.

By transforming the above formula, the selected NPT parameters can be advantageously selected. For example, low NPT pressure values are obtained by increasing the radius or by reducing the core layer thickness or by selecting the elastomer with a lower shear modulus value. The shaping of the length of the contact patch was discussed above in the paper (Section 3.1. Radial stiffness).

**Figure 10:**
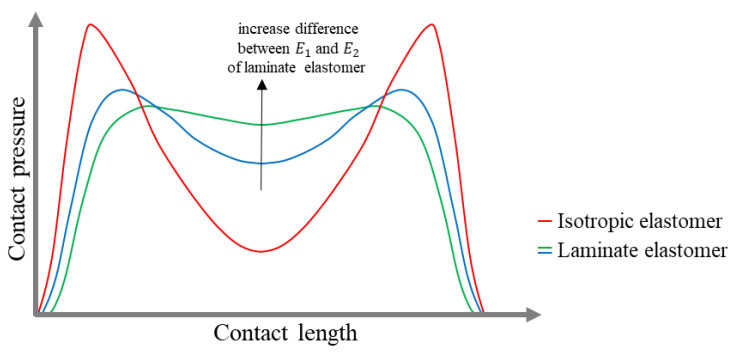
Contact pressure curves along the contact length of an unreinforced NPT band using an isotropic elastomer and a composite elastomer (different elastomers) (figure based on [13]).

In [13], the contact pressure curve along the length of the NPTs’ contact path was presented, in which unreinforced cores made of isotropic elastomer (red line) and a composite band (blue and green lines) were used (Figure 10). The isotropic elastomer band was characterized by stress concentration at the ends of the contact path. The composite band consisted of several elastomer layers (laminate elstomer), one of which had a Young’s modulus ($E_{1}$) of at least 150 MPa, while the other elastomer had a value of Young’s modulus ($E_{2}$) not exceeding 30 MPa. By using elastomers with different Young’s moduli in the band, a uniform pressure distribution was obtained in the contact patch (green line). The pressure becomes progressively more uniform as the difference between $E_{1}$ and $E_{2}$ increases (this tendency was marked by a black arrow in Figure 10). A uniform pressure distribution along the length of the NPT contact patch is obtained when the ratio of band deflection under shear to band deflection under bending is high. In a homogeneous unreinforced band with through holes, this is achieved by increasing the mass moment of inertia, which is achieved by increasing the distance between the through holes [14].

In [40], the results of numerical research concerning, among others, the pressures in the contact patch of NPT with a hexagonal elastic structure on a non-deformable ground, the band of which was modelled from two inextensible steel membranes with a polyurethane core, were presented. Stress concentrations were obtained in the central part of the rectangular contact patch. A band of a similar structure was modelled in the research [41]. Different types of cellular elastic structures were analyzed. Stress concentration in the central part of the rectangular contact area was observed for vertical loads of up to 1500 N. Increasing the load caused pressure concentration at the beginning and end of the contact area. In [43], the influence of the 2D and 3D cellular structure on the pressures in the contact patch was analyzed. The NPT was modeled with a uniform band reinforced with two inextensible membranes. Circumferential grooves were modeled in the tread area. The NPT with a 3D structure was characterized by higher pressures, which could result from the places where the cell walls were connected to the band. In [44], the influence of the elastic structure on the contact pressures was presented. The applied bottom loader-type elastic structure caused pressure concentration at the place of connection with the tread/band. Two angular positions of the NPT were also analyzed, which resulted in obtaining different positions of the structure compressed under the wheel axle. This allowed for the visualization of the formation of contact pressures depending on the position of the elastic structure, the concentration of which occurred at the place of connection of the structure with the tread. In [42], an elastic structure with single spokes was proposed, the shape of which imitated the upper limb of a kangaroo. Regardless of the load, the elastic structure was characterized by a significant concentration of pressures in the middle of the contact length.

The results of experimental research on the NPT’s contact patch with non-deformable ground can be found in [6,45,46,47]. Experimental research on the interaction of NPT with deformable ground can be found in [48]. In [45], a comparative analysis of the contact patch of NPT and pneumatic tires, which are designed for skid-steer loaders, was made. The studies were performed for three different values of vertical load. The length of the contact patch of the pneumatic tire decreased with the increase of the inflation pressure. At each of the analyzed contact pressures, the pneumatic tire was characterized by a longer contact length. The width of the NPT trace changed slightly, which resulted mainly from the shape of the tread area. The analysis of the contact patch of wheels designed for UTVs (Utility Terrain Vehicles) is presented in [6]. The studies used NPTs with a spoke and cellular elastic structure and radial and diagonal pneumatic tires. NPT with a cellular elastic structure was characterized by the highest average contact pressures, which resulted from the small contact area with the non-deformable ground and low density of the tread pattern. The NPT with an elastic spoke structure had a mean value of contact pressures similar to a diagonal tire.

In [46], the results of model and experimental research of NPT with a 2D cellular structure were presented. A rectangular contact area was obtained, and the contact length was changed under the influence of load. The results confirm the observations presented in Figure 9a.

In [49], 2D cellular structures were analyzed for NPT contact pressures. Hexagonal (“honeycomb”) structures were analyzed in which one was created by connecting hexagonal cells, while the other was created by cutting circles at a specific diameter, which was characterized by thicker connection points compared to the honeycomb structure. Increasing the cell density (number of cells and layers) results in an increase in radial stiffness and an increase in NPT load-bearing capacity. NPT equipped with hexagonal cells was characterized by a higher load-bearing capacity with the same number of cells compared to an elastic structure with cut circles. Increasing the density of the elastic structure caused a decrease in contact area and contact pressures.

In [48], the interaction of NPT with deformable ground was analyzed. Experimental research was conducted for NPT with the original and reduced number of spokes. Reducing the number of spokes resulted in a decrease in soil compaction.

Similar to PTs, the contact path of NPTs depends significantly on the normal load. Moreover, experimental studies have confirmed that a change in the contact patch occurs with longitudinal force loading [29].

Quick summary:

- Contact pressure is one of the design criteria for NPTs. It increases with the thickness and shear modulus of the shear band, but decreases as the NPT radius increases.
- The design of the shear band with the tread significantly affects the dimensions of the contact patch. NPTs with an “arc” tread area allow for increased contact patch width as the vertical load increases. However, NPTs with a “flat” tread area do not exhibit this behavior.
- Using the laminated elastomer core and the unreinforced band allows for more even pressure distribution across the contact patch. In contrast, the isotropic elastomer shear band concentrates pressure at the beginning and end of the contact patch.
- The NPT contact patch area changes under the influence of vertical load, while in a typical pneumatic tire, the inflation pressure significantly affects the contact patch area.

### 3.5. Rolling Resistance

During NPT rolling or driving at a steady speed on an undeformable ground, cyclic band deformation occurs (flattening the band creates a contact path). The associated hysteresis losses account for approximately 90–95% of total losses [50]. The paper [45] compared the hysteresis-induced component of the rolling resistance force of an NPT and a pneumatic tire. The calculations were made based on the results of experimental research, which included determining the radial characteristic and measuring the contact patch. The rolling resistance component was calculated using the equation [51]:

(2) $$F_{tH}=\frac{w_{H}F_{N}zb}{A_{W}}$$

$F_{tH}$—component of the rolling resistance force resulting from wheel deflection,
$w_{H}$—hysteresis coefficient,
$F_{N}$—vertical force acting on the wheel axle,
z—wheel’s static deflection under the load of force $F_{N}$,
b—contact width,
$A_{W}$—actual contact area.

The tested NPT was characterized by higher radial stiffness than the pneumatic tire. At the same value of vertical load, the NPT had less deflection. The flat tread area of NPT influenced the invariance of the contact width regardless of the load. The NPT, regardless of the load, was also characterized by a hysteresis coefficient twice as high as the tested pneumatic tire. For vertical loads of 4 kN and 12 kN, the calculated value of the NPT rolling resistance force was comparable to the results of the pneumatic tire. An increase in the rolling resistance force of the NPT by 40% compared to the pneumatic tire was observed for a normal load of 20 kN.

In [17], the hysteresis loop areas of the NPT’s elastic structure equipped with single spokes, cellular structure, and 3D structure were compared. The 3D structure used was characterized by the largest volume, which resulted in the largest energy losses during NPT vertical deflection. The type of structure used will affect the energy losses during cyclic deformation. With the increase in the volume of the elastic structure material, the energy losses increase.

In numerical research, the rolling resistance force can be calculated indirectly from the energy losses inside the NPT. The energy losses can be calculated from the strain energy, the material loss tangent, and the NPT volume, while the rolling resistance force is calculated by dividing the energy losses by the tire circumference length [52]:

(3) $$S_{NPT}=\int_{v}S_{E}tan\left(\delta \right)v$$

(4) $$F_{t}=\frac{S_{NPT}}{L_{NPT}}$$

$S_{NPT}$—the NPT’s loss energy,
$S_{E}$—the strain energy,
tan(δ)—hysteresis coefficient (the phase lag between stress and strain in the material of the shear beam),
v—the total NPT volume,
$L_{NPT}$—the NPT circumference length.

The rolling resistance force can also be estimated at the band design stage. For a uniform core, the following relation can be used [19]:

(5) $$F_{t}≅\tau_{max}\gamma_{max}\tan\left(\delta \right)h$$

$\tau_{max}\;$—shear stress for the maximum shear angle of the core material,
$\gamma_{max}$—maximum shear angle of the core material,
tan(δ)—hysteresis coefficient (the phase lag between stress and strain in the material of the shear beam),
h—shear beam core thickness.

One of the methods of achieving low rolling resistance is to use materials with low hysteresis (e.g., steel, aluminum) as the band. Another approach is to use porous elastomers reinforced with fibers, e.g., carbon fiber [53]. In [54], an analysis of the influence of changing the spoke thickness and the modulus of shear deformation of the band made of polyurethane was carried out using the finite element method. Changing the thickness of the band from 10 to 20 mm caused a reduction in rolling resistance by approximately 32%, an increase in radial stiffness by approximately 63%, and an increase in the maximum contact pressure by approximately 27%. Changing the shear deformation modulus from 6 MPa to 20 MPa caused an almost linear increase in radial stiffness by approximately 234%, a reduction in rolling resistance by approximately 59%, and an increase in the maximum contact pressure by approximately 85%. Changing the spoke thickness from 3 mm to 5 mm resulted in a slight reduction in rolling resistance (i.e., by approx. 18%), an increase in radial stiffness by approx. 70%, and a slight increase in the maximum contact pressure, i.e., by approx. 7%.

Quick summary:

- The materials used for the band and the elastic structure directly impact the rolling resistance of NPTs. Using materials with low hysteresis loss positively impacts the reduction in rolling resistance.
- The rolling resistance of NPTs is reduced by:
  ○ decreasing the thickness of the flexible structure components (e.g., reducing the thickness of the spokes),
  ○ decreasing the thickness of the banding,
  ○ increasing the shear deformation modulus of the band (which reduces the contact patch and may cause an increase in contact pressures).

## 4. The Use of NPTs in Vehicles

The use of NPTs in wheeled vehicles is not a new idea. Press information about NPT fitted with a ribbed rubber spokes design dates back to 1938 [55]. The biggest advantages of modern NPTs are replacing the properties of pneumatic tires’ compressed air and their resistance to punctures. Vehicles using NPTs are not exposed to failures related to the loss of compressed air. In pneumatic tires, the air pressure should be controlled, which will affect, e.g., rolling resistance and contact pressures. NPTs are maintenance-free in this area. The disadvantage of modern NPTs is the inability to change the NPT contact area, which can make it difficult to drive in particularly rough terrain. NPTs are used in applications where a pneumatic tire could be easily damaged, causing the vehicle to be immobilized.

One such area is vehicles designed for underground mining. The additional advantage is the ability to drain water from the contact patch into the NPT, minimizing the possibility of aquaplaning. In [56], two NPT prototypes were presented, designed for underground loaders used in mines. The proposed NPTs were tested up to 10 t. The designed NPTs can be equipped with side covers protecting their interior from water and stones.

NPTs can also find wide application in military vehicles, which are to provide protection to the crew, minimizing situations of risk to their lives, such as the need to replace a damaged tire during a combat mission. One of the key problems of military unmanned ground vehicles (UGVs) is the reliability of mobility, including tires [57]. A damaged pneumatic tire of an unmanned vehicle can be the cause of the failure of the UGV mission and the need to engage a human to repair the vehicle and expose it to enemy fire. NPTs are used to solve this problem because they are more resistant to damage than pneumatic tires, thus ensuring the success of the UGV mission.

The advantages of NPT were noticed by the South African Army, whose pneumatic tires of the Rooikat armored reconnaissance vehicle, due to frequent damage, constituted a significant part of the operating costs [58]. Terra Trak Puncture proof tire was tested in Rooikat vehicles, and its advantages, apart from eliminating compressed air, included flame retardancy, acid, and UV resistance. Another important area of military vehicles is the threat associated with improvised explosive devices. Open spaces of the elastic structure do not constitute a “barrier” to the overpressure wave created by the explosion.

Key tire manufacturers are developing various NPT designs that have potential applications in a wide range of vehicles. Michelin has proposed the X^®^ Tweel™ Airless Radial Tire Family [59]. The NPTs with 2D elastic structure are equipped with radially arranged spokes. The manufacturer offers NPTs for Zero Turn Radius Mowers, Skid Steers and Truck-Mounted Forklifts, All Terrain Vehicles and Utility Terrain Vehicles, Golf Carts. There are also some attempts to use NPTs in unmanned vehicles [57]. The MICHELIN^®^ Uptis (Unique Puncture-Proof Tire System) is a solution designed for passenger cars. Michelin and General Motors have entered into cooperation covering research on the possibility of using UPTIS in passenger vehicle models from 2024 [60]. The acquisition of Resilient Technologies™ by Polaris Defense enabled the use of NPT technology [61]. Polaris Defense developed Terrain Armor Non-Pneumatic Tires, which feature a flexible 2D hexagonal elastic structure. The NPTs are offered for use by customers in civilian vehicles [62]. Goodyear developed NPT to support autonomous urban transportation at the Jacksonville Transportation Authority. NPT was tested in Olli autonomous vehicles [63]. Goodyear expanded testing of its airless tires to high-performance electric vehicles (Tesla Model 3) traveling at highway speeds [64]. It is worth noting that electric vehicles have high torque values that are applied to the wheels. In 2015, Hancook proposed the NPT i-Flex for passenger cars [65]. In 2022, a new i-Flex model was proposed for UGV by Hyundai Rotem [66]. In [67], an NPT made using 3D printing technology was presented, which is intended for a prototype two-wheeled electric vehicle. The possibility of using large-format printing in NPT production can help to reduce the environmental impact of transporting tires from manufacturer to consumer.

In [68], an NPT designed for a vehicle weighing 1000 kg with a mechanism enabling the change in its outer diameter was presented. The NPT consisted of steel flat springs, rubber wheel feet, a screw transmission mechanism (change in outer diameter), and outer and inner hubs. The advantage of this solution is the possibility of adjusting the wheel diameter and the length of the contact patch in order to ensure optimal conditions on rigid and primitive roads.

**Figure 11:**
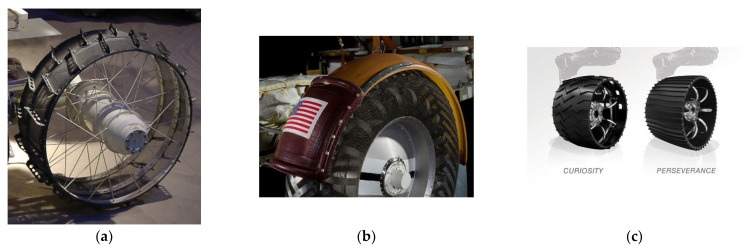
NPTs used in space missions: (**a**) NPT of the Lunokhod vehicle *, (**b**) NPT of Lunar Roving Vehicle **, (**c**) NPT of the Mars rovers *** (* photo taken from [70] under the CC 3.0 licence—photo has been cropped, ** photo taken from [71] CC0 under the license, *** photo taken from [72]—this file is in the public domain in the United States because it was solely created by NASA).

NPTs have also been used in missions to explore the Moon and Mars [65,69]. Ensuring vehicle mobility requires meeting the challenges of large daily temperature changes; no or different atmosphere than on Earth; lower gravity compared to Earth; exposure to solar radiation; and operation in a vacuum. Large temperature changes require the use of resistant materials. The Soviet Union’s unmanned remotely controlled vehicle Lunokhod (Moon Walker in English) was sent to the Moon in 1970 (on the Luna17 starship) and in 1973 (on the Luna 21 starship). The vehicle was equipped with eight rigid wheels. The wheels were made of rims to which a net was attached and cleats connected to the hub with bicycle spokes (Figure 11a).

The NPT used in the NASA (National Aeronautics and Space Administration) Lunar Roving Vehicle resembled pneumatic tires in their shape. The manned vehicle was equipped with four flexible wheels (Figure 11b) made of wire mesh [73]. In the part corresponding to the tread, steel strips were attached to ensure traction properties on the lunar soil. Detailed information on the development of NPTs intended for lunar exploration is presented in [73]. Curiosity Rover (2012) and Perseverance Rover (2020) were used for Mars exploration. The wheels were made of aluminum alloys. Based on the Curiosity Rover’s experience with wear resulting from interaction with Mars rocks, narrower wheels with a larger diameter were made for the Perseverance Rover. The tread blocks (cleats) were also changed, i.e., their number was increased and a milder profile was used (instead of chevron-patterned) (Figure 11c) [72].

Table 1 compares selected functional properties of NPTs and PTs. The following properties were selected for comparison: air pressure, puncture risk, maintenance, and weight. Cost comparisons were not performed because, in addition to operating costs, product lifecycle costs, including production and disposal, must be considered.

Quick summary:

- NPTs are used in vehicles where standard pneumatic tires can be damaged, causing the vehicle to become immobilized, e.g., vehicles used in mines and military vehicles.
- Their use in UGVs is very promising, as it allows the vehicle to be used in difficult conditions without the need for human intervention to improve the flat tire.
- The ability of NPTs to drain water (e.g., through holes in the tire’s sidewall) can prevent aquaplaning.
- The greater mass of NPTs, as well as their mass moment of inertia, compared to typical pneumatic tires, results in greater resistance to changes in rotational speed, such as acceleration and braking.

## 5. Conclusions

NPTs are interesting products that can be used in a wide range of vehicles. Their radial stiffness is mainly due to the applied elastic structure. Optimal load support is obtained when the elastic structure has high effective radial stiffness in tension and low effective radial stiffness in compression. For NPTs equipped with single spokes (NPT top loaders), the radial characteristic will be shaped by the appropriate selection of spoke length/curvature. The radial stiffness of the NPT bottom loader (in most cases equipped with a cellular elastic structure) depends on the type of cells used and the internal angles describing the cells. Regardless of the structure used, it is important to ensure its susceptibility to buckling, which will protect against stresses in the structure.

Tangential stiffness (longitudinal and lateral) of the wheel affects vehicle behavior during curvilinear motion. The susceptibility of the NPT elastic structure to circumferential deformations shapes the longitudinal stiffness of NPT. The elastic cellular structure will be characterized by smaller circumferential belt displacements, which will translate into greater longitudinal stiffness compared to NPT equipped with single radial spokes. In most NPTs, it is not possible to select the side walls that occur in pneumatic tires. The 2D elastic structure will have significant resistance to lateral deformation of the NPT, which will affect the lateral stiffness of the NPT. A promising solution is the use of a 3D flexible structure, which allows for greater lateral displacements.

The contact path depends on the shear beam/band of NPT, which mimics the properties of compressed air. The parameters of the contact patch, i.e., width and length, depend on, among others, the shape of the tread area. The NPT with a flat tread area will be characterized by an almost constant width with increasing vertical load. The opposite phenomenon will be observed for NPTs with an arc tread area. The paper presents a mathematical relationship that allows for designing contact pressures based on the selected geometric dimensions and selection of the NPT material. The uniformity of pressures through contact length will be ensured by a shear beam with a laminate elastomer core.

Another important issue is the energy losses of rotating NPT. The review has shown that increasing the volume of the elastic structure will result in increased energy losses and rolling resistance.

The ability to replicate the properties of pneumatic tires without the need to maintain compressed air allows NPTs to be used in vehicles operating in harsh conditions. Military applications in unmanned land vehicles are particularly interesting. Damage to a pneumatic tire during a mission often requires human intervention to repair the vehicle. The use of NPTs eliminates (to a certain extent) the risk of vehicle immobilization due to tire damage. Despite the fact that NPTs never get flat, it is necessary to periodically check the tread wear, the condition of the elastic structure, and the condition of the adhesive bond between the individual components. The possibility of retreading will significantly extend the life of NPTs.

## Figures and Tables

**Table 1:** Comparison of selected functional properties of NPTs and PTs [6,74,75,76,77].

	Non-Pneumatic Tires	Pneumatic Tires
Air pressure	The properties of compressed air are mimicked by the shear band. The possibility of eliminating compressed air allows NPTs to be used in difficult terrain conditions. However, the lack of air pressure restricts changes to the radial characteristics or contact patch during use.	Compressed air at a specific pressure is necessary for the proper use of a typical pneumatic tire. Changing the pressure allows you to shape the directional characteristics (radial, longitudinal, and lateral), contact pressure, and change the contact path required, e.g., in the field. However, tire damage and loss of compressed air prevent further use of the vehicle, which results, e.g., in increased operating costs and makes the military vehicle an easy target.
Puncture risk	The risk of punctures was eliminated. NPT can be used with a partially cracked elastic structure and partial loss of adhesion between the rim and elastic structure and shear beam. Cracks in the elastic structure can be repaired and reused. Possibility of retreading.	Hitting a sharp object, curb, or pothole can cause damage. Using the tire in difficult terrain increases the risk of tire damage. The possibility of using run-on-flat solutions partially solves these problems, but increases wheel weight and costs.
Maintenance	Extensive damage to the elastic structure may render the NPT unusable. It is necessary to inspect and repair (prevent further propagation) cracks in the elastic structure in order to extend the service life of NPTs. Loss of adhesion between the rim, elastic structure, and shear beam may occur. Removing objects (e.g., stones) that are stuck in the elastic structure will extend its service life.	In order to operate the vehicle in optimal conditions, it is necessary to check the correct inflation pressure. Periodically check pneumatic tires for signs of wear or damage to the tread and sidewall, such as bubbles, cuts, or punctures. Using the wrong tire size for the rim will cause accelerated wear.
Weight	The band, being a significantly thick element, is distant from the wheel axis, which negatively affects the mass moment of inertia. Consequently, there is greater resistance to rotational movement, especially during acceleration and braking. Greater unsprung mass.	The lower weight of the wheels will have a positive effect on the chassis and the load on the suspension system.

## Data Availability

No new data were created or analyzed in this study. Data sharing is not applicable to this article.
